# Thigh pyomyositis caused by group A streptococcus in an immunocompetent adult without any cause

**DOI:** 10.1186/s13104-016-2346-2

**Published:** 2017-01-07

**Authors:** Kensuke Minami, Tsuneaki Kenzaka, Ayako Kumabe, Masami Matsumura

**Affiliations:** 1 Division of General Internal Medicine, Jichi Medical University Hospital, Shimotsuke, Japan; 2 Department of General Medicine, Toyooka Public Hospital, Toyooka, Japan; 3 Division of Community Medicine and Career Development, Kobe University Graduate School of Medicine, 2-1-5 Arata-cho, Hyogo-ku, Kobe, Hyogo 652-0032 Japan

**Keywords:** Pyomyositis, Group A streptococcus, Immunocompetent adult

## Abstract

**Background:**

Pyomyositis is typically caused by *Staphylococcus aureus*, and is rare in temperate climates, although its prevalence has been recently increasing. This infection often involves the thigh, and is associated with immunodeficiency.

**Case presentation:**

We report the case of a healthy 20-year-old Japanese woman who experienced a fever and continuous pain for several days. She was admitted to our hospital and was diagnosed with pyomyositis after we discovered an abscess between the muscles of her dorsal distal left thigh using computed tomography. This is a rare case of thigh pyomyositis, as it was caused by group A streptococcus and occurred in an immunocompetent adult from a temperate climate.

**Conclusions:**

Our review of the literature revealed that group A streptococcus pyomyositis typically occurs in temperate climates, among young adults without any underlying disease, and is associated with a poorer prognosis, compared to general pyomyositis. We suggest that pyomyositis should be considered when immunocompetent adults present with apparently idiopathic inflammatory muscle lesions.

## Background

Pyomyositis is a bacterial infection of the skeletal muscle that is most commonly caused by *Staphylococcus aureus* (*S. aureus*; 90% of tropical cases and 75% of temperate cases) [1], although it is relatively rare in temperate climates. Another 1–5% of cases involve group A streptococcus, and other rare cases may involve streptococcus (groups B, C, and G), pneumococcus, *Neisseria* spp., *Haemophilus* spp., *Aeromonas* spp., *Serratia* spp., *Yersinia* spp., *Pseudomonas* spp., *Klebsiella* spp., and *Escherichia* spp. [1]. The development of pyomyositis is typically associated with immune-related conditions (e.g., diabetes mellitus, the use of corticosteroids, human immunodeficiency virus (HIV) infection, acquired immune deficiency syndrome (AIDS), and malignancies), and most commonly affects the muscles of the lower extremities (i.e., the thighs and calves) [2]. Among immunocompetent patients, pyomyositis is typically accompanied by a history of a traffic accident [3] or vigorous exercise [4]. Furthermore, pyomyositis caused by group A streptococcus (GAS) is a known complication of chicken pox among children [5]. Therefore, we report a case of apparently idiopathic thigh pyomyositis that was caused by GAS in an immunocompetent adult.

## Case presentation

A 20-year-old Japanese woman was working in a restaurant and experienced discomfort in her left thigh on the seventh day before her hospital admission. On the next day, she felt pain in the same area while walking, and subsequently consulted a physician, who prescribed an antiphlogistic sedative patch on the fifth day before her admission. On the third day before her admission, she developed a fever (38 °C) and subsequently consulted another physician, who prescribed an antipyretic drug. However, she continued to experience fever and pain in her left thigh flexor during rest. Therefore, she consulted another physician and was admitted to our hospital, where she was prescribed a single dose of acetaminophen (400 mg). She did not have a history of domestic or overseas travel, participation in outdoor activities, or trauma.

**Fig. 1:**
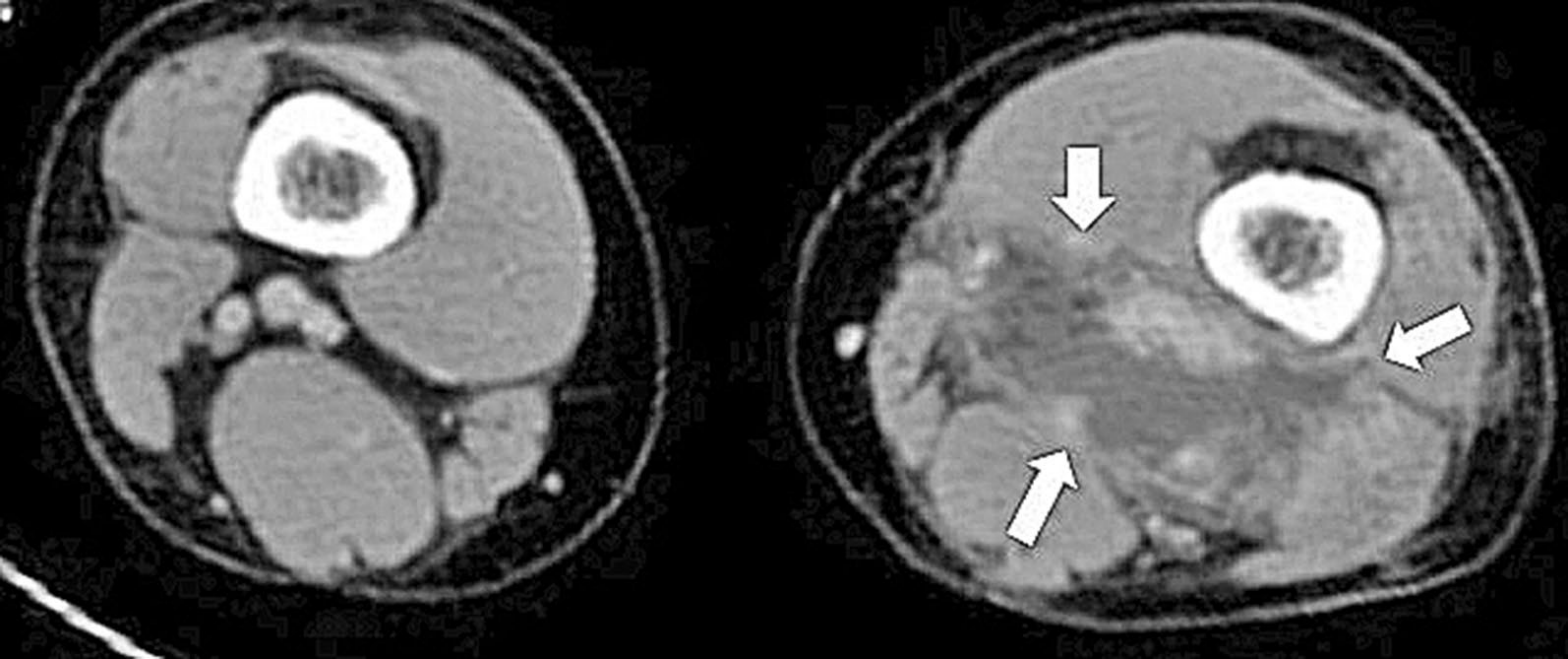
Enhanced computed tomography reveals fluid between the dorsal distal muscles of the *left thigh* and an enhanced margin of fluid (*white arrows*)

Upon admission, she had a blood pressure of 110/63 mmHg, a pulse of 107 beats/min, a respiratory rate of 16 breaths/min, and a temperature of 38.9 °C. Her left thigh flexor appeared slightly red, with mild swelling, warmth, and pain. However, her head, lungs, heart, abdomen, and back did not exhibit redness or swelling. Her white blood cell count was 12,100 cells/mm^3^, her hemoglobin level was 10.8 g/dL, and her C-reactive protein level was 24.11 mg/dL. Her liver and renal functions were normal, and the HIV antibody/p24 antigen test (fourth generation) provided negative results. However, enhanced computed tomography (CT) revealed fluid between the dorsal distal muscles of her left thigh and an enhanced margin of fluid (Fig. 1), which we diagnosed as an abscess. There were no other abscess-like formations outside of her lower extremities.

An incision was made in her left thigh on the day of admission, the abscess was continuously drained, and we treated her using ampicillin/sulbactam (four doses per day, up to 12 g daily). Two sets of blood cultures provided negative results; however, a culture of a fasciotomy specimen from the abscess revealed positive results for GAS. Definitive identification of the GAS isolate was performed using the rapid streptococcus test (Rapid ID32 STREP; bioMerieux SA, Marcy l’Etoile, France). Therefore, we changed the patient’s treatment to penicillin G (24 million units per day via continuous infusion) on day 5. However, drug eruption in response to the penicillin G was suspected on day 12, and we changed the antibiotic to clindamycin on day 13 (2700 mg in three doses per day). We stopped the continuous drainage on day 15, and switched the treatment to oral clindamycin on day 16 (600 mg three times per day). The patient was discharged after 17 days in the hospital, and her antibiotic treatment lasted 4 weeks. We did not observe any side effects that were related to the clindamycin treatment. The patient has remained in good health during the 1-year follow-up after her discharge from the hospital.

## Conclusions

We encountered a rare and apparently idiopathic case of non-tropical pyomyositis that was caused by GAS in a healthy young Japanese woman. In this context, pyomyositis is rare in temperate climates, although it has recently become more frequent in these climates [6]. The clinical features of temperate and tropical cases of pyomyositis are summarized in Table 1.

**Table 1:** The general clinical features of pyomyositis in temperate and tropical cases

	Temperate cases	Tropical cases
Ages [1, 2, 7]	Adults (elderly)	Children (2–5 years) and adults (35–40 years)
Underlying conditions [2, 8]	Immunocompromised or serious underlying conditions, such as HIV infection, diabetes mellitus, leukemia, chronic renal failure, asplenia, scleroderma, rheumatoid arthritis, Felty’s syndrome, chemotherapy, or immunosuppressive treatment	Healthy
Microbiology (*S. aureus*) [1] (%)	60–75	>90
Positive blood cultures [1] (%)	20–30	5–10
Mortality [6, 9] (%)	6.0–9.4	0.5–2.0

The clinical features of the reported GAS pyomyositis cases are summarized in Table 2 [5, 9–27]. Many of these cases occurred in temperate climates (20/24, 83.3%), and most patients were children or young adults, with relatively few middle-aged patients (mean age, 30.3 ± 21.0 years). However, the 20 cases of GAS pyomyositis from temperate climates exhibited a mean age of 31.5 ± 22.3 years, and only included six pediatric cases, which differs from the general characteristics of tropical pyomyositis. Furthermore, GAS infection is a known complication of chicken pox in children [5], although only 1 of the 6 pediatric cases was GAS-positive. Although pyomyositis typically occurs in the lower extremities (9/24, 37.5%) [5, 9–27], other muscle groups can be involved, such as the iliac [10], psoas [11, 17], iliopsoas [11], trunk [5], neck [13, 18, 26, 27], and upper extremity muscles [14, 16, 19, 22, 25]. Involvement of multiple muscle groups is also common (9/24, 37.5%). Interestingly, cases of GAS pyomyositis typically resemble general pyomyositis [28], although many cases are apparently idiopathic, which is more similar to cases of tropical pyomyositis [2]. Positive blood cultures were observed in 9 of the 24 temperate cases (37.5%), approximately 60% (14/24) of the cases involved surgical drainage (which was performed via fasciotomy), and no cases required amputation. The mortality rate was 12.5% (3/24), and four cases involved an intensive care unit stay, which would indicate that the temperate cases were more severe than general pyomyositis [6, 9]. Thus, GAS pyomyositis typically occurs in temperate climates, among young adults without underlying disease, and is associated with a poorer prognosis, compared to general pyomyositis. The reasons for these observations are unclear, although *Mandell, Douglas, and Bennett’s Principles and Practice of Infectious Diseases* [29] indicates that most GAS pyomyositis cases occur spontaneously or after blunt non-penetrating trauma, and the bacteria are most likely hematogenously translocated from the throat to the deep tissues. In addition, GAS is the most common cause of bacterial pharyngitis among children and young adults [29]. Furthermore, children and young adults are often highly active and may readily experience blunt non-penetrating trauma. Therefore, we hypothesize that the clinical features of GAS pyomyositis may be related to these factors.

**Table 2:** Clinical features of group A streptococcus pyomyositis

Age (years)	Sex	Tropical	Underlying condition	Area of infection	Single or multiple lesions	Blood cultures	ICU stay	Antibiotics	Treatment duration (days)	Drainage	Prognosis	Reference
2	M	No	None	Left thigh	–	Positive	–	2nd cef, PCG, aminoglycoside	40	Surgical	Recovered	[9]
2	M	No	None	Right iliac muscle	Single	Negative	No	–	21	No	Recovered	[10]
4	M	No	Autism	Left psoas/thigh	Multiple	Positive	Yes	CTRX, VCM → PCG, CLDM	–	Surgical	Recovered	[11]
5	F	No	None	Right thigh	Single	Negative	No	Oxacillin	–	Surgical	Recovered	[12]
8	M	No	None	Neck	–	–	–	–	–	–	–	[13]
9	M	No	None	Right iliopsoas	Multiple	Positive	No	–	33	Surgical	Recovered	[11]
17	M	Yes	–	–	Multiple	Positive	–	–	–	–	Recovered	[14]
19	M	No	None	Right thigh and left knee	Multiple	–	No	PCG	–	Surgical	Recovered	[15]
23	M	Yes	–	Left arm	Single	Negative	–	–	–	–	Recovered	[14]
24	F	No	None	Right neck	Single	Negative	No	CEZ	28	Surgical	Recovered	[27]
26	F	No	None	Left thoraco-abdominal muscle	Multiple	Negative	Yes	Ticarcillin-clavulanate → PCG	7	Surgical	Recovered	[5]
30	F	No	None	Left hand and both thighs	Multiple	Positive	Yes	CTRX, CLDM	–	–	Recovered	[16]
31	M	Yes	None	Left psoas	–	Negative	–	PCG	–	No	Recovered	[17]
33	F	No	DM	Left axilla	Single	Negative	No	PCG	20	Surgical	Recovered	[17]
38	F	No	None	Right shoulder/arm	Multiple	Negative	No	PCG	11	Surgical	Recovered	[19]
40	F	No	None	–	Multiple	Positive	–	–	–	–	Died	[20]
44	F	No	None	Left calf/knee	Multiple	–	–	CTX, metro, PCG → PCG	–	Surgical	Recovered	[21]
48	M	No	None	Left neck	Single	Negative	No	Ticarcillin-clavulanate, CLDM → PCG	28	Surgical	Recovered	[18]
49	F	No	None	Right arm	–	–	–	PCG	–	–	Died	[22]
52	M	No	None	Left thigh	Single	–	No	Cloxacillin, PCG	6	Surgical	Recovered	[23]
53	M	No	None	Left calf	–	Positive	–	PCG, aminoglycoside	–	–	Died	[20]
67	M	No	None	Left thigh	–	–	–	CEZ → PCG	10	Surgical	Recovered	[24]
76	M	No	IPF	Right biceps	Single	Positive	Yes	PCG	31	Surgical	Recovered	[25]
–	F	Yes	None	Neck	Single	Positive	No	Flucloxacillin, PCG	–	No	Recovered	[26]

*M* male; *F* female; *ICU* intensive care unit;– not recorded; → switch; 2nd cef second-generation cephalosporin; *PCG* benzylpenicillin; *CTRX* ceftriaxone; *VCM* vancomycin; *CLDM* clindamycin; *CTX* cefotaxime; metro, metronidazole; *CEZ* cefazolin; *DM* diabetes mellitus; *IPF* idiopathic pulmonary fibrosis

It is difficult to diagnose pyomyositis, as there are no specific symptoms, and the only early symptoms are minor pain and swelling. Therefore, patients are often slow to consult with a physician, and the diagnosis is often delayed by 2–3 weeks [1]. When the diagnosis is delayed, death can be caused by septic complications, which include toxic shock syndrome [9]. In the present case, the patient exhibited minor swelling, redness, and warmth in her thigh, and we were able to rapidly diagnose pyomyositis based on our physical findings and the CT results. This rapid diagnosis allowed us to quickly start antibiotic treatment and drainage, which are both critical for treating pyomyositis (except during the early stage) [30].

We reported a rare case of apparently idiopathic thigh pyomyositis that was caused by GAS in an immunocompetent adult. Furthermore, our review of the literature revealed that GAS pyomyositis typically occurs in temperate climates, among young adults without any underlying disease, and is associated with a poorer prognosis, compared to general pyomyositis. Therefore, pyomyositis should be considered when a patient presents with inflammatory lesions in the thigh or other common pyomyositis sites, even when the patient is not immunocompromised and does not exhibit the standard characteristics of pyomyositis.

## Abbreviations

GAS: group A streptococcus
CT: computed tomography
HIV: human immunodeficiency virus
AIDS: acquired immune deficiency syndrome
